# How Does Hospital Microbiota Contribute to Healthcare-Associated Infections?

**DOI:** 10.3390/microorganisms11010192

**Published:** 2023-01-12

**Authors:** Flora Cruz-López, Adrián Martínez-Meléndez, Elvira Garza-González

**Affiliations:** 1 Subdirección Académica de Químico Farmacéutico Biólogo, Facultad de Ciencias Químicas, Universidad Autónoma de Nuevo León, Pedro de Alba S/N, Ciudad Universitaria, San Nicolás de los Garza 66450, Nuevo León, Mexico; 2 Laboratorio de Microbiología Molecular, Departamento de Bioquímica y Medicina Molecular, Facultad de Medicina/Hospital Universitario “Dr. José Eleuterio González”, Universidad Autónoma de Nuevo León, Avenida Gonzalitos y Madero s/n, Colonia Mitras Centro, Monterrey 64460, Nuevo León, Mexico

**Keywords:** healthcare-associated infections, hospital microbiota, infection control, antimicrobial resistance, surfaces persistence

## Abstract

Healthcare-associated infections (HAIs) are still a global public health concern, associated with high mortality and increased by the phenomenon of antimicrobial resistance. Causative agents of HAIs are commonly found in the hospital environment and are monitored in epidemiological surveillance programs; however, the hospital environment is a potential reservoir for pathogenic microbial strains where microorganisms may persist on medical equipment surfaces, on the environment surrounding patients, and on corporal surfaces of patients and healthcare workers (HCWs). The characterization of hospital microbiota may provide knowledge regarding the relatedness between commensal and pathogenic microorganisms, their role in HAIs development, and the environmental conditions that favor its proliferation. This information may contribute to the effective control of the dissemination of pathogens and to improve infection control programs. In this review, we describe evidence of the contribution of hospital microbiota to HAI development and the role of environmental factors, antimicrobial resistance, and virulence factors of the microbial community in persistence on hospital surfaces.

## 1. Introduction

Prevention of healthcare-associated infections (HAIs) has been a subject of continuous research due to associated high costs, high mortality and morbidity rates, and the emergence of drug-resistant pathogens as causative agents [1]. The U.S. Centers for Disease Control and Prevention (CDC) estimates that 5% of all hospital admissions result in a HAI, culminating in approximately 722,000 infections and 75,000 deaths each year, with a burden of $28–33 billion in costs (https://epi.dph.ncdhhs.gov/cd/hai/figures.html, accessed on 15 October 2022). Diverse strategies have been applied to reduce or control infections in the hospital environment, and these efforts are focused on eliminating microbial sources. However, HAIs remain a threat to health and human life even in developed countries [2]; for example, in the USA, 1 in every 31 patients develop at least one HAI every day [2].

HAI development has been associated with inadequate antimicrobial prescription or consumption, insertion of medical devices, concomitant diseases, immunological status of patients, and adherence to hygiene practices [3,4]. In addition, exposure to microorganisms from a nosocomial environment may contribute to HAI development [5]. The microbiota is a set of microorganisms in a particular environment and their interactions [6]; its composition is prone to vary in time and scale. The most frequent bacteria found in indoor environments include *Pseudomonas* spp., *Acinetobacter* spp., *Staphylococcus* spp., *Corynebacterium* spp., *Sphingomonas* spp., and *Clostridium* spp., while frequent fungi found are *Aspergillus* spp., *Penicillium* spp., and *Cladosporium* spp. [7].

The importance of the microbial communities in a hospital resides in its contribution to health or disease in patients; however, this aspect is not well understood [6]. The risk of pathogen acquisition is higher in a hospital environment [8]; thus, recognition of pathogen reservoirs in hospital settings, transference of microorganisms between the external environment and patient microbiota, and other interactions must be considered for infection control strategies. The aim of this review is to describe evidence of the contribution of the hospital microbiota to HAI development and the role of environmental factors, antimicrobial resistance, and virulence factors of the microbial community in persistence on hospital surfaces.

## 2. Microbial Communities in a Hospital Environment

Based on microbiological and sequencing techniques, numerous studies have described microbiota profiles from hospital environments. Microbial profiles vary according to hospital settings, as shown in Table 1; various studies have shown the presence of bacteria associated with skin and soil on hospital surfaces, including offices and highly touched restroom surfaces [5,9,10,11,12]. 

According to Cruz-López et al., bedrails are one of the most contaminated surfaces near patients and, among medical devices, mechanical ventilation tubes are one of the most colonized surfaces in patients that developed ventilator-associated pneumonia, between day 1 and day 3 at the step-down care units (SDCUs) [5,10]. In this study, commensal microorganisms decreased over time, and a high diversity of gram negative was observed on day 8 on all related surfaces to patients. Additionally, high microbial diversity was observed among patients’ relatives over time, including coagulase-negative staphylococci (CoNS), *Acinetobacter baumannii*, *Klebsiella pneumoniae*, *Klebsiella aerogenes*, *Stenotrophomonas maltophilia*, and *Enterococcus faecalis.* In addition, *A. baumannii*, *Enterobacter cloacae*, *K. pneumoniae*, *Pseudomonas* spp., *Raoultella ornithinolytica*, and *Staphylococcus aureus* were recovered from ten nurses [10].

Some studies have suggested that hospital microbiota has a homogeneous structure due to an overlap observed among the bacterial communities present in the facilities analyzed [13]. Similarly, Yano et al. observed that the population structure in almost all locations of one of the analyzed hospitals (hospital C) and some locations in the other hospitals formed a cluster, being very similar with Enterobacteriaceae as the predominant family [14]. Authors suggested that the unconcerned harmful behaviors of nurses while treating patient wastes or cleaning sinks shared by patients or visitors resulted in the spread of bacteria originating from patients or sinks to hospital wards. This factor may help to explain variability in the relationship between hospital contamination and HAIs [14].

Chopyk et al. analyzed the temporal variations in a bacterial community of an adult ICU prior to closing for renovations, during the renovation process, and after re-opening, showing the impact of microbiota from patients and healthcare workers (HCWs). In this study, environmental bacteria predominated after closure and human-associated bacteria prevailed after re-opening and before closure. *Bacillaceae*, *Cutibacterium*, *Streptococcus*, *Ralstonia*, *Herbaspirillum*, and *Staphylococcus* were present during all study stages on the bedrail. Four core taxa were shared among the keyboard, the sink, and the bedrail throughout the study, including *Bacillaceae*, *Cutibacterium*, *Herbaspirillum*, and *Ralstonia*. *Staphylococcus*, *Streptococcus*, and *Cutibacterium* (human-associated bacteria) were dominant core microbiota on bedrails during the “before closure“ stage. Conversely, *Staphylococcus* and *Streptococcus* abundance in samples collected during “after closure” decreased and were eventually exceeded by *Bacillaceae* in the “before opening” stage. Human-associated bacteria increased in abundance in samples of “after opening” stage, mainly *Cutibacterium* [12].

Importance about the composition of microbial communities is that it may harbor opportunistic pathogens able to establish an infection in vulnerable subjects under conditions, such as disruptions of the skin and mucosal barriers and gut microbiota dysbiosis. For example, *Clostridioides difficile* inhabiting the gastrointestinal tract may proliferate after prolonged consumption of extended-spectrum antibiotics; antibiotic consumption is associated with the loss of gut microbiota members, which favors *C. difficile* expansion [15]. 

Skin disruption after a traumatic event (wounds or insertion of invasive devices) may facilitate the colonization and subsequent infection by opportunistic pathogens harbored in the microbiota or microorganisms from the environment [16,17,18]. The microbiota characterization of chronic wounds revealed that *Staphylococcus* spp. (63%) and *Pseudomonas* spp. (25%) were most frequent genera detected in an analysis of 2963 samples [19]. *S. aureus* and *S. epidermidis* were the most prevalent species, and methicillin-resistant *Staphylococcus* species were present in 741 out of 2963 samples (25%) from chronic wounds. Moreover, *Corynebacterium* spp., *Propionibacterium* spp., and anaerobic bacteria were highly frequent in chronic wounds [19]. 

In addition, it has been reported that constituting microorganisms of the chronic wound microbiota are commonly organized into biofilms [18]. James et al. reported the presence of biofilms in 60% of chronic wounds (30 out of 50 samples, *p* < 0.001), and in 6% (1 out of 16 samples) of acute wounds [20]. According to this study, *Staphylococcus* spp. and *Enterococcus* spp. were the predominant genera in sampled wounds, although Gram-negative bacilli, such as *Pseudomonas* spp. and *Proteus* spp., were also present [20]. In addition, biofilms are prevalent (78.2%) in nonhealing chronic wounds, which may contribute to chronic wound persistence and deficient wound healing [21].

In addition, patients’ microbiota act as a source of causative agents of infections. In a study where 198 catheters from different anatomical sites were analyzed, 47 (23.7%) were contaminated with heterogeneous microbiota. In 37 out of 47 catheters (78.7%) CoNS were detected, and in 10 out of 47 samples (21.3%) pathogenic microorganisms were found, such as *E. coli* (7.8%), *Enterobacter* spp. (4.5%), *Klebsiella* spp. (4.5%), *Morganella morganii* (1.5%), and *P. aeruginosa* (1.5%) [22]. 

Probably, a reason for these reported variations is the study method employed. Some studies have described the microbial composition of hospital surfaces and the dynamics of colonization on corporal surfaces of patients and inanimate surfaces based on culture. However, the main limitation associated with this technique is the inability to recover non-viable and non-cultivable microorganisms. This limitation can be overcome by applying next-generation sequencing (NGS)-based methods, including ITS and 16S rRNA gene sequencing and shotgun metagenomic sequencing. NGS-based studies provide new insights into hospital microbiome and colonization processes. First, ITS and 16S rRNA gene sequencing methods allows us to describe the diversity of fungi and bacteria (respectively) by comparing phylogeny and taxonomy from complex microbiomes or environments [23,24]. Second, shotgun metagenomic sequencing has allowed the characterization genes associated with drug resistance and virulence factors in microbial communities [25]. In addition, NGS-based methods allow the detection of genetic material from non-viable cells or non-cultivable microorganisms in vitro [25].

Multiple parameters can alter NGS data; among these, sample collection and processing can cause most of the variability. NGS studies have been crucial for tracking and identifying the origins of persistent multidrug-resistant pathogens to eradicate the infection source and prevent hospital dissemination [26]. However, the application of NGS data in the clinical practice is still in development for the surveillance or tracking pathogens [27]. Surveillance studies are difficult to perform on a routine basis and require detailed individual-level patient clinical data to reveal the transfer process of microorganisms and even the transfer of genetic material within microbial communities on environmental surfaces in hospitals. The current studies reveal an area of opportunity for the understanding of disease development, progression, diagnosis, and therapy.

## 3. Hospital Microbiota Sources

It has been observed that the microbiota of corporal surfaces from HCWs is similar to the microbial communities present in hospital environments, due to the dynamics and interaction of HCWs between facilities within the hospital [11]. Therefore, patients’ microbiota can also be a source of microorganisms in the hospital environment or the contamination of high-touch surfaces [5,13]. Furthermore, 20–60% of HAIs are associated with direct contact of HCWs with patients; thus, HCWs act as a vector for pathogen transmission or even as reservoirs for cross-transmission, as shown in Table 2 [28,29]. 

Infected patients also act as a source of pathogens; a high proportion of HAIs is a consequence of patient-to-patient transmission and surfaces close to patients are frequently contaminated with HAI-associated pathogens (Table 2) [5,30]. These contaminated surfaces lead to the transmission of pathogens in the hospital environment [31]. Diverse studies have reported that an infection with *Pseudomonas aeruginosa*, *A. baumannii*, or *C. difficile* in the previous occupant of a room is a risk factor for the acquisition of the same infection by subsequent occupants [32,33,34]. 

**Table 2:** Vectors of transmission in hospital facilities.

Author	Unit/ Ward	Traced Pathogen	Evidence of Pathogens Transmission	Ref.
Agodi et al.	ICU	*P. aeruginosa*	One-hundred and thirty-eight isolates were recovered from 45 patients; 61% of the isolates were highly genetically related and were distributed in 46% of patients. Cross-transmission was observed in 59% of the colonized or infected patients.	[29]
Hassan et al.	Adult and pediatric medicine.	*K. pneumoniae*	The most frequently recovered from surfaces near patients infected (bedsheet, towel, bedrail, etc.) by this species.	[30]
Weber et al.	Non- specified	*C. difficile*	*C. difficile* spores have been found in the rooms of infected patients by this pathogen (2.9% to 75%) and have been isolated from the hands of infected patients and the hands of HCWs in charge of these patients.	[28]
Saughnessy et al.	ICU	*C. difficile*	Up to 11% of the patients who acquired CDI after admission had a prior occupant with CDI (*p* = 0.002).	[32]
Nseir et al.	ICU	*P. aeruginosa*, *A. baumannii*	The prior occupant with a multidrug-resistant *P. aeruginosa* or multidrug-resistant *A. baumannii* strain in an ICU room is an independent factor for its acquisition by a subsequent patient (OR 2.3, 95% CI 1.2–4.3, *p* 0.012; and OR 4.2, 95% CI 2–8.8, *p* < 0.001, respectively).	[34]
Cruz-López et al.	SDCU	*A. baumannii*, *K. pneumoniae*, *E. cloacae*	Genetically related isolates associated with the causative agents of HAIs were widely distributed on inanimate surfaces near patients and in the skin folds of patients and medical devices inserted before and after infection outcome. *A. baumannii* clones (associated with HAI development in patients) were detected in 4/35 nurses who attended units analyzed.	[5]

CDI: *Clostridoides difficile* infection, HAIs: healthcare-associated infections, HCW: healthcare workers, ICU: intensive care units, SDCUs: step-down care units.

The presence of microorganisms on high-touch surfaces may represent transitory contamination or contamination that remains stable over time [13]. These surfaces are recognized as reservoirs of potentially infectious agents that facilitate their dissemination despite cleaning procedures [13]. Often, the causative agents of HAIs are found on surfaces near patients, such as tables, bedrails, monitors, privacy curtains, and ventilator buttons [5,10,11]. Moreover, they are found on stethoscopes and invasive devices, such as central venous catheters and endotracheal tubes [5,5,8,10].

The presence of Gram-negative strains genetically related to HAI causative agents, recovered before and after the HAI outcome, have been reported; these microorganisms persisted on environmental surfaces despite disinfectants and dryness conditions [5]. Distinctively, species such as *K. pneumoniae* and *E. cloacae* were recovered after HAI development; these pathogens were isolated for the first time from clinical samples, and recovery was gradual in other environmental surfaces [5]. 

## 4. Environmental Factors

The indoor environmental conditions such as temperature, seasonal trend, built design, ventilation, occupancy, inhabitants, and the operational characteristics of buildings influence on the microbiota composition of an environment [11,35,36]. Some examples are included in Table 3. 

The occupants of a building are an important factor in shaping their microbial community. High occupancy increases the accumulation of human-related microorganisms and the potential transmission of microorganisms through increased social interactions, contact with surfaces (direct or indirect), and air flow (Table 3) [7]. Diverse studies suggest that resuspended floor dust is an important source of bacterial aerosol populations during occupancy [37]. 

**Table 3:** Effect of environmental conditions on microbial composition of hospital surfaces.

Environmental Factor	Effect on Microbial Composition	Ref.
Higher temperatures/higher luminance	Greater microbial dissimilarity between patient and surface microbial communities within a patient room on the same day.	[11]
Higher luminance	Reduction of human associated bacterial communities and inactivation of viruses in indoor surface	[36]
High relative humidity and humidity ratio	Significant microbial similarities in a hospital room on the same day.	[11]
High relative humidity	Improved persistence of bacteria, i.e., *Acinetobacter* spp. survives for 11 days at ≥31% relative humidity and 4 days at 10% relative humidity.	[38]
Seasonal trend	The microbiota from nose and hands of different HCWs on the same floor are highly similar during late summer/early fall. In contrast, they are least similar in the winter.	[11]
Seasonal trend	BSIs caused by Gram-negative microorganisms were more frequently reported during the summer period than during winter.	[39]
Higher humidity	Significant similarity in microbial community of the hand and nose among different HCWs.	[11]
Higher temperature	Less similarity in microbial community of the hand and nose among different HCWs.	[11]
Higher temperature	Each 5.6 °C increase in the mean temperature was related to a rise in the frequency of BSIs by Gram-negative bacteria (independent of season).	[39]
High occupancy	Elevated concentrations of indoor airborne microorganisms. Increased bacterial genome concentration in indoor air and floor dust.	[7,37]

In addition, floors are an important reservoir of human-associated microbiota due the high concentration of bacteria specific to the hair, nostrils, and skin of humans found in floor dust and indoor air. Furthermore, the particle shedding of desquamated skin cells and their subsequent resuspension strongly influenced the airborne bacteria population structure in this human-occupied environment. The inhalation of microbes shed by other current or previous human occupants may occur in communal indoor environments [37]. The indoor microbial communities found in dust and surfaces contain microbial populations from the human skin, gut, oral, and urogenital microbiomes [7]. Moreover, occupant-associated microbiomes may disperse in spaces by direct human contact, aerosols and particulates from human surfaces; and indoor dust containing microorganisms from human microbiomes [7]. 

## 5. Bacterial Virulence Factors and Environmental Persistence

Most of the described interactions in the hospital environment are among the microbiota patients, the microbiota of HCWs, and the hospital microbiota [11]. The skin of patients and the skin of HCWs probably determine the composition of the microbial community on hospital surfaces over time by the transference of microorganisms [10,11]. 

Virulence factors expressed by bacteria allow them to colonize host niches and to contribute to virulence and pathogenesis [40]. Bacterial virulence factors may play a role in the stability of hospital microbiota. Microbial species have diverse virulence factors, such as proteolytic enzymes, extracellular capsules, lipopolysaccharides, outer membrane proteins, and biofilms [40,41]. The hospital environment is hostile to microorganisms, owing to the use of antimicrobial agents, disinfectant solutions, and cleaning protocols and adverse changes in temperature and humidity; thus, the expression of virulence factors is influenced by environmental stimuli for physiological adaptation [41].

The extracellular capsule (the outermost layer of a bacterial cell) allows microorganisms to withstand environmental perturbations, such as desiccation and antimicrobial agents. This structure is frequently found in environmental bacteria and clinical isolates; moreover, it may contribute to the colonization of patients and pathogenesis [42]. Biofilms are structures that confer resistance to disinfectant solutions, detergents, desiccation, and antimicrobial agents. Biofilms comprise multiple species and remain attached to inanimate surfaces [43,44]. Hu et al. determined that biofilms may persist for up to 1 year on frequently cleaned surfaces and are detected on approximately 93% of the studied surfaces in an ICU [43]. In addition, spores contribute to the environmental persistence of clinically relevant pathogens, such as *C. difficile*. Spores provide resistance against desiccation and cleaning products on hospital surfaces and medical equipment over the years [45].

However, it is important to understand that hospital microbiomes contribute to HAI development via the transmission of genetic material associated with antimicrobial resistance, virulence, and persistence between pathogens and commensal microorganisms in clinical settings. Moreover, mobile genetic elements (MGEs) contribute to worsening outbreaks by transferring certain traits, such as the ability to form biofilms or virulence characteristics. In this regard, biofilms are known to increase the probability of plasmid transfer carrying genes associated with virulence factors and persistence; however, this process is restrained to subpopulations that do not initiate a cascade of horizontal plasmid spread, due to physicochemical and biological factors within biofilms. Prior studies have shown that MGEs can potentially exacerbate outbreaks; unfortunately, techniques that allow the tracking of the movement of MGEs are limited. Bacterial whole-genome sequencing (WGS) has modified the understanding of the epidemiology of drug-resistance and virulence genes through the identification of plasmid replicons, transposases, etc. [27].

## 6. Antimicrobial Resistance and Hospital Microbiota

Antimicrobial resistance is a phenomenon that further complicates HAI development owing to the presence of resistant pathogens, which leads to reduced therapeutic options [2]. The spread of antimicrobial-resistant microorganisms is favored by antibiotic misuse in animal feed as prophylaxis, an inability to complete prescribed therapeutic regimens, and poor adherence to antimicrobial stewardship programs for the administration and prescription of broad-spectrum or novel antibiotics [2]. 

Antibiotic resistance genes are abundant in both pathogenic and non-pathogenic microorganisms in the hospital microbiota; thus, the hospital microbiota may function as a reservoir for antimicrobial resistance genes [2,11]. Antibiotic-resistant microorganisms may persist on hospital surfaces for long periods and may be asymptomatically carried by HCWs, who interact with patients [2]. 

Identifying the hospital resistome may allow us to establish systematic evaluation to handle resources for preventing infections [46]. Chang et al. described a phylogenetic study to characterize the microbiome and antibiotic resistance determinants on surfaces related to 45 patient beds in a tertiary-care hospital. They followed 179 sites for 16 months and collected 358 samples, which were analyzed by deep shotgun metagenomic. Analysis revealed multidrug-resistant strains widely distributed colonizing across sites, as well as diverse environmental niches of microorganisms and antibiotic resistance genes associated with human microbiota and biofilm producer microorganisms. In this study, the authors recovered 63 Mb of phage and 696 Mb of plasmid sequences, most of which are not present in existing databases, pointing out the unrevealed genetic diversity in hospital environments. In addition, researchers noted the presence of gene combinations not previously seen; for example, large *mecA*-carrying plasmids that harbored disinfectant or antiseptic resistance genes (*qacA* or *qacC*), and plasmids with several antimicrobial resistance gene classes, such as *aac6-Aph2*, *dfrC*, and *lnuA*. Moreover, the authors suggest that clinical isolates can persist in hospitals for extended periods (>8 years), which allows them to opportunistically infect patients [46]. 

The colonization process with multidrug-resistant microorganisms is a significant risk factor for HAI development [47]. The relevance of multidrug resistance in the hospital environment is the exchange of resistance genes in MGEs between bacterial species, such as plasmids, insertion sequences (IS), transposons, gene cassettes, integrons, and genomic islands [48]. MGEs carrying antibiotic resistance genes often encode other determinants related to increased virulence and environmental persistence, which can be shared or exchanged between microbiota members [49]. This exchange of genetic material in the hospital environment may turn non-pathogenic bacteria into antibiotic-resistant bacteria. Thus, via horizontal gene transfer, microorganisms develop a resistance to several antibiotics and disinfectants at once [49].

Recently, Evans et al. screened the genomes of 2173 bacterial isolates using whole genome sequencing. Isolates were recovered from HAIs in one hospital over 18 months, and the presence of 10 plasmids carrying antimicrobial resistance genes in isolates of different species or the same species but another sequence type were detected, which suggest the transmission of MGE only without the pathogen transmission [49]. Silke et al. demonstrated the transfer of a 40 kb plasmid carrying the *bla*IMP-8 gene among the isolates of *P. aeruginosa* and *Citrobacter freundii* and its evolution to a 164 kb plasmid, which was subsequently transferred to the *Citrobacter cronae* isolates responsible for outbreaks at the same hospital in 2009 and 2012 [50]. These results provide evidence for the transfer of genetic determinants independent of bacterial transmission.

In some studies, certain antimicrobial resistance genes (ARG) have been detected in both hospital surfaces and in patients. Haverkate et al. reported the rate of blaOXA-48 transfer between *K. pneumoniae* and *E. coli* within hosts using epidemiological data and mathematical modeling [51]. Furthermore, at the community level, *Shigella* outbreaks, due to the transfer of a plasmid harboring an azithromycin resistance gene, have been reported in the United Kingdom. The latter allowed the spreading of the pathogen since traditional antibiotics had no activity against *Shigella* [52]. 

Similar evolutionary processes facilitate the transfer of genetic material in clinical environments; however, in clinical settings, this transfer often has fatal results, particularly for immunocompromised patients.

## 7. Aspects to Consider for Infection Control

**Figure 1:**
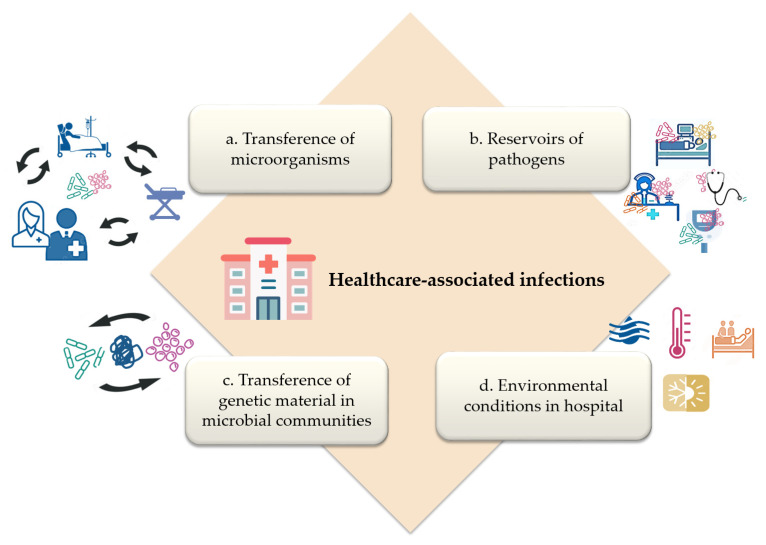
Factors related to healthcare-associated infections development. (**a**) transference of microorganisms between patients, healthcare workers, and hospital environmental surfaces; (**b**) hospital environmental surfaces, medical devices, healthcare workers, and patients as reservoirs of pathogens; (**c**) transference of genetic material associated with virulence, persistence, and antibiotic resistance in microbial communities; (**d**) environmental conditions, such as temperature, seasonal trend, humidity relative, and occupancy support persistence, diversity, and abundance of microorganisms on environmental surfaces.

Identifying resident microorganisms at each hospital facility may help to track potential pathogens during outbreaks. As previously mentioned, the hospital microbiota represents a source of contamination that may be associated with infection in vulnerable patients [2]. It has been shown that pathogens prevail in hospital environments, despite adverse conditions, such as low humidity, variations in temperature, and the use of disinfectants and antimicrobial agents [11,36]. Virulence factors expressed by microorganisms and their antimicrobial resistance mechanisms make them a severe threat in the hospital environment; in addition, genes associated with virulence and antimicrobial resistance could be transferred horizontally within a microbial community [49]. These factors, along with microorganism transfer among patients, HCWs, and environmental surfaces are related to HAI development (Figure 1), and they must be considered to propose new strategies in the control of HAIs.

The application of NGS-based methods allows us to demonstrate the horizontal transfer of genetic material between microorganisms through conjugation; however, transformation and transduction also can disseminate the resistance and virulence genes [27]. The conditions for transformation and transduction can be found in clinical settings; however, to date, there are few available tools to detect these mechanisms. The processes of transduction and transformation have gone undetected due to low rates and the difficulty of identification of recombination events. Furthermore, horizontal gene transfer generates a series of gene transmission events that modify phylogenetic relatedness between strains; thus, complicating the tracking and vigilance [27]. The diversity of genes associated with virulence and antibiotic resistance remarks the importance of continuous studies using molecular tools to track their mobility [27,49].

The determination of temporal patterns may suggest how microbial spread and interactions between microorganisms in the hospital environment occur [13]. Thus, detecting microbial communities in the hospital environment and tracking their composition changes along with tracking individual nosocomial pathogens may be useful for the design of strategies to reduce or prevent HAI development.

In addition, it is necessary to collect epidemiological data that, along with sequencing data, allows us to obtain clues during hospital microbiome characterization, pathogen tracking, infection control, and prevent the persistence of potential pathogens. Probably, the cost of these study methods, the time and knowledge required to work with sequencing data, and the routine implementation of the surveillance or tracking of pathogenic microorganisms hinder getting a complete puzzle until today.

## 8. Conclusions

For years, infection control programs have focused on the epidemiological surveillance of microorganisms recognized as pathogens, both on biotic and inanimate surfaces; however, infections continue to develop. The characterization of hospital microbiota may improve the understanding of the relationship between commensal and pathogenic microorganisms and their changes, due to variations in environmental factors. Thus, understanding the microbial community composition in the hospital environment may help in detecting variations that facilitate infection development, including predicting the risk of outbreaks or the emergence of resistant pathogens. These efforts could be the piece that, along with antibiotic stewardship programs, epidemiological surveillance, isolation rooms, surface cleaning, disinfection programs, and adherence to hand hygiene protocols, among other actions, will provide efficient approaches to prevent infections from drug-resistant pathogens.

## Figures and Tables

**Table 1:** Sources of infection in hospital facilities.

Author	Predominant Microorganisms	Source of Infection	Unit or Ward	Ref.
Hewitt et al.	*Staphylococcus*, *Streptococcus*, *Neisseria*, *Enterobacter*, *Pseudomonas*, *Acinetobacter*, *Clostridium*, *Fusobacterium*, *Gemella*, *Leclercia*, *Propionibacterium*, *Corynebacterium*, *Lactobacillus.*	Baby bedside, door button, incubator, sink, and weigh cart.	Neonatal ICU	[9]
Rampelotto et al.	*Acinetobacter*, *Pseudomonas*, *Staphylococcus*, *Klebsiella*, *Streptococcus*.	Bedrail, table, chair, dispenser, water tap, serum and gas support, cabinets, surgical table, computer, infusion pumps, and others.	Surgery center, emergency department, medical unit, and ICU.	[13]
Lax et al.	*Acinetobacter* and *Pseudomonas*, before building inauguration. *Corynebacterium*, *Staphylococcus*, and *Streptococcus*, after HCW and patients´ introduction.	Floor, bedrail, staff pager, computer, countertop, and others. Corporal surfaces from patients and HCW.	Ten patient rooms and two nursing stations from hematology and oncology wards.	[11]
Cruz-López et al.	Coagulase negative staphylococci, *Acinetobacter baumannii*, *Enterococcus faecalis*, and *Klebsiella pneumoniae*.	Bedrail and table near patients. Medical devices inserted in patients. Corporal surfaces from patients, relatives, and HCW.	Step-down care units.	[10]
Yano et al.	Enterobacteriaceae	Patient overbed table, patient room table, nurse desk, nurse wagon, patient room skin.	Surgical and internal wards, from three hospitals.	[14]
Copyk et al.	*Burkholderiaceae*, *Bacillaceae*, and *Rhizobiaceae* during “before opening” stage. *Corynebacteriaceae*, *Staphylococcaceae*, *Streptococcaceae*, *Acinetobacter*, *Bacteroides*, *Pseudomonas*, and *Enterobacteriaceae* “before closure” stage.	Bedrails, computer keyboards, and sinks.	Adult ICU	[12]

HCW: healthcare workers, ICU: intensive care units.

## Data Availability

Not applicable.
